# *Giardia intestinalis* mitosomes undergo synchronized fission but not fusion and are constitutively associated with the endoplasmic reticulum

**DOI:** 10.1186/s12915-017-0361-y

**Published:** 2017-04-03

**Authors:** Luboš Voleman, Vladimíra Najdrová, Ásgeir Ástvaldsson, Pavla Tůmová, Elin Einarsson, Zdeněk Švindrych, Guy M. Hagen, Jan Tachezy, Staffan G. Svärd, Pavel Doležal

**Affiliations:** 1 Department of Parasitology, Faculty of Science, Charles University, Průmyslová 595, Vestec, 252 42 Czech Republic; 2 Department of Cell and Molecular Biology, BMC, Uppsala University, Uppsala, Sweden; 3 Institute of Immunology and Microbiology, First Faculty of Medicine, Charles University and General University Hospital, Prague, Czech Republic; 4 Institute of Cellular Biology and Pathology, First Faculty of Medicine, Charles University, Prague, Czech Republic

## Abstract

**Background:**

Mitochondria of opisthokonts undergo permanent fission and fusion throughout the cell cycle. Here, we investigated the dynamics of the mitosomes, the simplest forms of mitochondria, in the anaerobic protist parasite *Giardia intestinalis*, a member of the Excavata supergroup of eukaryotes. The mitosomes have abandoned typical mitochondrial traits such as the mitochondrial genome and aerobic respiration and their single role known to date is the formation of iron–sulfur clusters.

**Results:**

In live experiments, no fusion events were observed between the mitosomes in *G. intestinalis.* Moreover, the organelles were highly prone to becoming heterogeneous. This suggests that fusion is either much less frequent or even absent in mitosome dynamics. Unlike in mitochondria, division of the mitosomes was absolutely synchronized and limited to mitosis. The association of the nuclear and the mitosomal division persisted during the encystation of the parasite. During the segregation of the divided mitosomes, the subset of the organelles between two *G. intestinalis* nuclei had a prominent role. Surprisingly, the sole dynamin-related protein of the parasite seemed not to be involved in mitosomal division. However, throughout the cell cycle, mitosomes associated with the endoplasmic reticulum (ER), although none of the known ER-tethering complexes was present. Instead, the ER–mitosome interface was occupied by the lipid metabolism enzyme long-chain acyl-CoA synthetase 4.

**Conclusions:**

This study provides the first report on the dynamics of mitosomes. We show that together with the loss of metabolic complexity of mitochondria, mitosomes of *G. intestinalis* have uniquely streamlined their dynamics by harmonizing their division with mitosis. We propose that this might be a strategy of *G. intestinalis* to maintain a stable number of organelles during cell propagation. The lack of mitosomal fusion may also be related to the secondary reduction of the organelles. However, as there are currently no reports on mitochondrial fusion in the whole Excavata supergroup, it is possible that the absence of mitochondrial fusion is an ancestral trait common to all excavates.

**Electronic supplementary material:**

The online version of this article (doi:10.1186/s12915-017-0361-y) contains supplementary material, which is available to authorized users.

## Background

The mitochondria of opisthokonts are dynamic cellular compartments that undergo constant fusion and division events [1]. These processes control mitochondrial morphology and ensure that the mitochondrial network remains homogenous across the cell [2].

GTPases from the dynamin superfamily have a central role in controlling mitochondrial dynamics. The division apparatus relies on the function of the soluble dynamin-related protein Drp1/Dnm1 [3], which is recruited to the mitochondrial surface by several membrane-anchored proteins, such as Fis1 and Mff [4, 5]. The opposing fusion processes require the membrane-anchored, dynamin-related proteins mitofusins/Fzo1 [6] and Opa1/Mgm1 [7] in the outer and inner mitochondrial membranes, respectively. However, information on the fusion and its apparatus is limited to animals and fungi. Whether mitochondria of other lineages of eukaryotes also fuse remains largely unknown.

Recent studies have shown the prominent role of the endoplasmic reticulum (ER) tubules in mitochondrial dynamics in fungal and mammalian cells [8–11]. Different molecular tethers between the ER and the mitochondria have been functionally described in both fungi [11–14] and mammalian cells [15], although for the latter the data have been questioned recently [16].

The transformation of endosymbiotic alphaproteobacteria into current-day mitochondria involved a redesign of their division apparatus. The bacterial divisome complex, which is built around the polymers of a tubulin ortholog, the GTPase FtsZ, has been entirely replaced in the mitochondria of many eukaryote lineages by proteins of the dynamin superfamily [17]; yet, eukaryotes that have preserved the original FtsZ-based machinery can still be found in all eukaryotic supergroups [18, 19].

Our detailed understanding of the molecular background of mitochondrial dynamics in opisthokonts is in sharp contrast to what is known about the rest of eukaryotic diversity. So far only a handful of eukaryotic species have been shown to employ dynamin-related proteins for mitochondrial division. Of the Excavata supergroup, which comprises a large collection of protist taxons, these include the parasitic kinetoplastid *Trypanosoma brucei* [20, 21] and the parabasalid *Trichomonas vaginalis,* the latter of which carries mitochondria-related organelles (MRO) known as hydrogenosomes [22]. Mitochondrial fusion has not been examined in any Excavata species so far, and neither have the orthologs of components of the fusion machinery been identified [23, 24].

Mitosomes represent the simplest form of MROs, which have independently arisen through convergent simplification in several protist lineages that inhabit oxygen-poor environments [25, 26]. While mitosomes have retained a double membrane, they have abandoned their mitochondrial genome and have dramatically reduced their proteome [27, 28].

*Giardia intestinalis* is an intestinal protist parasite of humans and other vertebrates and has been studied for a number of its unique cellular features, including the mitosomes [29, 30]. About 40–50 tiny mitosome vesicles are stably present in the active, motile stage of the parasite (trophozoite), with a prominent array of the organelles, referred to as central mitosomes, between the two nuclei of the trophozoite cell [31–33]. Mitosomes do not produce ATP, and their only identified metabolic role is in the formation of iron–sulfur clusters [29].

In this study, we investigated the dynamics of *G. intestinalis* mitosomes. We show that mitosomes are extremely steady organelles that do not fuse, and that their division is uniquely synchronized with mitosis. Mitosomes also divide in the encysting cell; thus, the infectious cyst contains two sets of organelles, which may facilitate rapid cytokinesis upon excystation in a newly infected host. Surprisingly, *G. intestinalis* mitosomes seems not to rely on dynamin-related protein during division but they associate with the ER throughout the cell cycle. The regions of contact between these two organelles are enriched for the lipid metabolic enzyme long-chain acyl-CoA synthetase 4 (LACS4), suggesting that the contacts define the sites of the lipid transport to the mitosomes.

## Results

### Mitosomes undergo neither fusion nor division during interphase

**Fig. 1:**
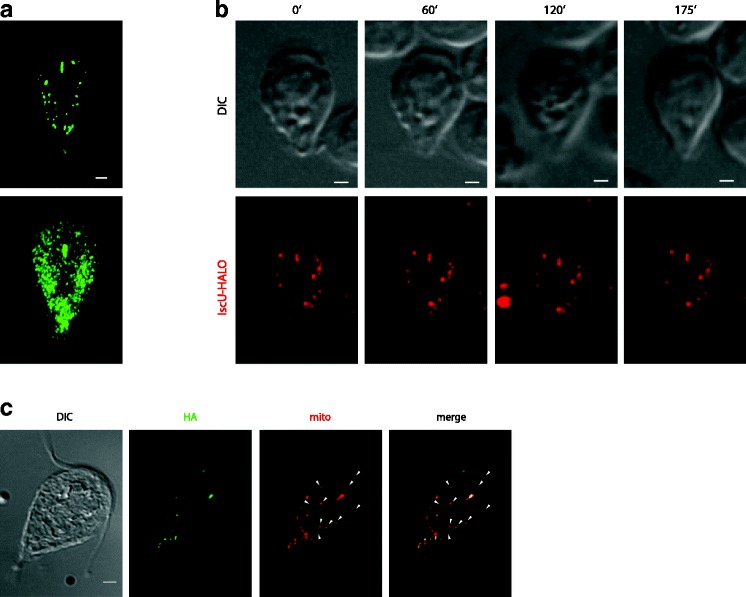
Mitosomes are stable organelles during interphase. **a** *G. intestinalis* trophozoites were fixed and immunolabeled with an anti-GL50803_9296 antibody. While the upper image shows a single *G. intestinalis* cell, the lower image represents the superposition of 25 imaged cells and shows areas of frequent and scarce mitosomal localization. **b** *G. intestinalis* cells expressing IscU-Halo were stained with the TMR Halo ligand and observed in medium containing 2% agarose under a confocal microscope equipped with a spinning disc. Still images (maximal projections of Z-stacks) from a time-lapse movie are shown with times indicated. Corresponding differential interference contrast (*DIC*) images are shown. Note that the number and distribution of organelles does not change during the indicated period of time. Scale bars, 2 μm. **c** *G. intestinalis* cells expressing human influenza hemagglutinin (*HA*)-tagged IscU were fixed and immunolabeled with an anti-GL50803_9296 antibody and anti-HA antibody. The *arrowheads* indicate mitosomes lacking the recombinant protein

The distribution of mitosomes in *G. intestinalis* trophozoites was followed using immunofluorescence and live-cell microscopy. As shown previously, each cell contains an array of multiple central mitosomes between the two nuclei and peripheral mitosomes that are spread throughout the cytoplasm (Fig. 1a). The superimposed images of multiple trophozoites showed that the mitosomes are plentiful at the lateral and posterior regions of the cell. Apart from between the two nuclei, the central region and the anterior end of the cell are devoid of mitosomes and low in mitosome number, respectively.

The live-cell fluorescence microscopy is hampered by weak fluorescence of green fluorescent protein (GFP) and its derivatives, which require the presence of oxygen to form the fluorescent tripeptide. Therefore, to follow the mitosomal dynamics in live cells, attached *G. intestinalis* trophozoites were observed using Halo-ligand-labeled mitosomal IscU [31]. The number of independent observations (e.g., Fig. 1b) showed no changes in the distribution or morphology of the organelles. This result suggests that mitosomes do not undergo division during interphase. Moreover, the lack of observable fusion among the mitosomes indicated that this behavior is either much less frequent or even absent in *G. intestinalis.*

**Fig. 2:**
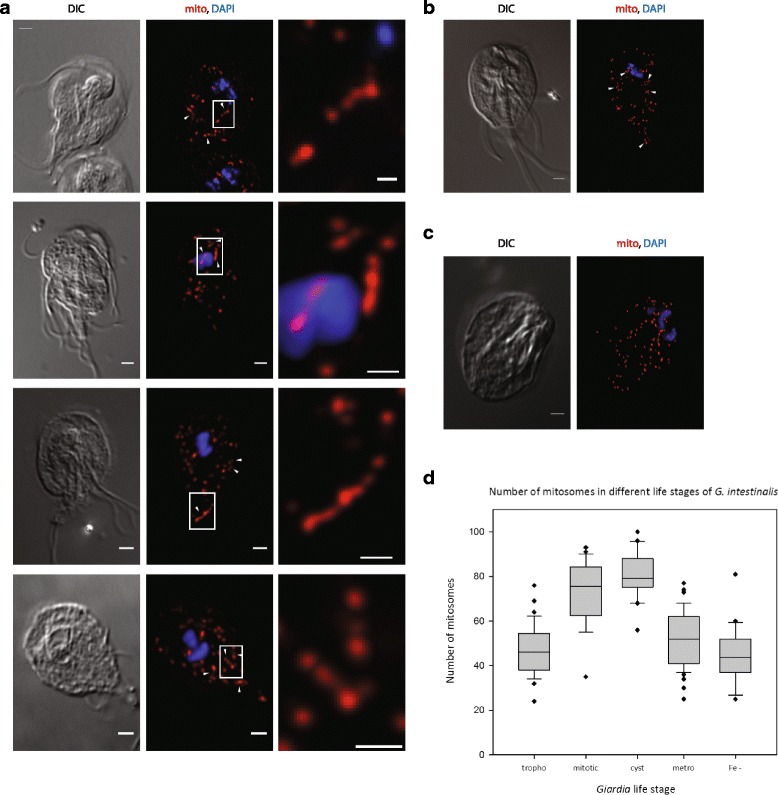
Mitosomes divide during mitosis. **a** A *G. intestinalis* culture was enriched for mitotic trophozoites by albendazole treatment (100 ng/ml) for 6 h at 37 °C. The cells were washed twice in warm medium and fixed, and the mitosomes were immunolabeled with an anti-GL50803_9296 antibody (*red*) and stained for nuclei with DAPI (*blue*). The image represents a deconvolved maximal projection of the Z-stack. Corresponding differential interference contrast (*DIC*) images are shown. Scale bar, 2 μm. An inset of the dividing organelle is shown on the *right. Arrowheads* indicate dumbbell-shaped dividing mitosomes. Scale bar, 0.5 μm. *G. intestinalis* cells were induced to encyst in vitro and the mitosomes were immunolabeled. **b** Encysting cell. **c** Completed cyst stage. **d** The mitosome numbers in *Girdia* cells in different cell/life stages, grown under metabolic stress (metronidazole/iron chelator). *tropho* trophozoite, *mitotic* mitotic cell, *cyst* cyst stage of *G. intestinalis*, *metro G. intestinalis* cells treated with 5 μM metronidazole, *Fe G. intestinalis* cells treated with 300 μM 2,2′-Bipyridyl (DIP). Statistical calculations were carried out using 30–50 cells in SigmaPlot. The *vertical lines* represent the mean values, the *gray boxes* depict the range in which 90% of the values fall, and the error bars depict the standard deviations. *Black dots* represent values outside the standard deviation range

We tested if the parasite responds to changes in metabolic conditions by varying the mitosome number by incubating cells in either iron-rich or iron-depleted media. The key proteins in *Giardia* energy metabolism, such as pyruvate:ferredoxin oxidoreductase (PFO) and 4Fe-4S ferredoxin, carry iron–sulfur clusters in their active sites [34]. Considering that synthesis of iron–sulfur clusters occurs exclusively in the mitosomes [29], up-regulation of the biosynthetic iron–sulfur cluster proteins [35] and an increased number of mitosomes could be expected to occur as a way to compensate for a lack of iron–sulfur proteins. However, no change in mitosome morphology or number was observed in iron-depleted cells (Fig. 2d).

Similarly, the cells were also grown with increasing concentrations of metronidazole, a 5-nitroimidazole antibiotic used to treat infections of anaerobic organisms including *Giardia* [36]. The compound is activated by electron transfer from low-redox-potential electron donors such as ferredoxins [37] and, for instance, induces morphological changes to hydrogenosomes of *Trichomonas vaginalis* [38, 39]. Mitosomes contain 2Fe-2S ferredoxin and are likely a place of metronidazole activation; however, the presence of metronidazole did not trigger any mitosome-related phenotype, even at lethal metronidazole concentrations (Fig. 2d).

### Mitosomal heterogeneity supports the lack of fusion

The absence of observable mitosomal fusions suggested that the organelles could exhibit some degree of heterogeneity. In animal and fungal cells, experimental abolition of mitochondrial fusion leads to fragmentation of the mitochondrial network and functional and morphological heterogeneity of the individual mitochondrial compartments [40, 41].

Uniformity of mitosomes was inspected by immunolocalization of the endogenous mitosomal protein GL50803_9296 [33] and the episomally encoded human influenza hemagglutinin (HA)-tagged mitosomal protein IscU. The fluorescence signals of both proteins co-localized to the same organelles in most instances but in every cell individual mitosomes were positive only for the endogenous protein (Fig. 1c). While the heterogeneity illustrates that the synthesis and/or the transport of the episomally expressed protein is not as efficient as that of the endogenous one, it also indicates that individual mitosomes did not fuse to homogenize their protein content.

### Mitosomes divide during mitosis

The lack of observable mitosomal division during interphase suggested that mitosomes might divide during mitosis. Live-cell microscopy of mitotic *G. intestinalis* cells is hampered by the rapid movement of the detached dividing cells, in which the adhesive disc depolymerizes. Nevertheless, observation of individual cells passing through mitosis indicated that mitosomes may divide during this stage of the cell cycle (Additional files 1, 2, 3 and 4). Thus, fixed *G. intestinalis* cultures enriched for mitotic cells were instead examined by immunofluorescence microscopy. To enrich the mitotic cells, starvation [42] as well as albendazole-dependent [43] methods were used. While both methods provided the same results concerning mitosomal dynamics, the latter was used owing to a higher degree of synchrony.


Additional file 2: Movie of the dividing *Giardia –* IscU-Halo. (AVI 5339 kb)
Additional file 3: Movie of the dividing *Giardia –* DIC. (AVI 4615 kb)
Additional file 4: Movie of the dividing *Giardia –* merged channels. (AVI 11947 kb)


In contrast to interphase cells, mitotic cells were found to contain a variety of elongated dividing mitosomes whose morphology ranged from dumbbell-shaped to thread-like structures (Fig. 2a). Importantly, these mitosomes were found across the whole cytoplasm and were usually dumbbell-shaped, which is a typical configuration for dividing vesicular structures [44–46]. This observation suggests that the individual mitosomes undergo independent and synchronized divisions during mitosis.

**Fig. 3:**
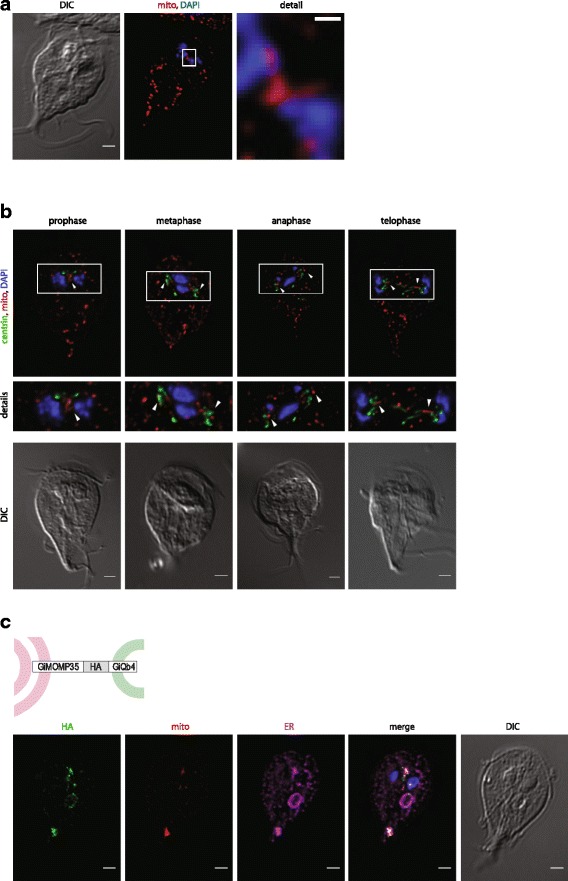
Central mitosomes divide during prophase and associate with *G. intestinalis* karyomastigont. **a** Mitosomes of mitotic cells were immunolabeled with an anti-GL50803_9296 antibody. The division of the central mitosome could be observed only during prophase as a prominent V-shaped arrangement before the segregation of the daughter kinetosomes and chromosomes. **b** *G. intestinalis* cells expressing C-terminally human influenza hemagglutinin (*HA*)-tagged centrin were enriched for mitotic cells by albendazole treatment. The cells were fixed and immunolabeled with anti-HA (*green*) and anti-GL50803_9296 antibodies (*red*). The nuclei were stained with DAPI (*blue*). *Arrowheads* indicate separation of the central mitosomes coupled with division of the basal bodies. Scale bars, 2 μm. **c** Expression of the synthetic linker composed of the outer mitosomal membrane protein GiMOMP35 and GiQb4 SNARE protein induces aggregation of the mitosomes to the cell periphery and the formation of the endoplasmic reticulum–mitosome chimeras. The cells were fixed and immunolabeled with anti-HA (*green*), anti-GL50803_9296 antibodies (*red*), and anti-PDI2 antibodies (*magenta*). The nuclei were stained with DAPI (*blue*). *DIC* differential interference contrast microscopy

Mitotic *G. intestinalis* cells were further studied to identify a possible connection between mitosomal division and a particular phase of mitosis. The dividing organelles were found during all phases of mitosis (Fig. 3b, Additional file 5) with the number of mitosomes gradually increasing toward telophase (Additional file 5). The only exception was the central mitosomes (Fig. 3a). The division of the central organelles, which are arranged as an array localized closely to the basal bodies [47], occurred exclusively in prophase, before the basal bodies moved toward the opposite spindle poles [48] (Fig. 3b). Sister arrays of mitosomes were often positioned to form a V-shaped structure (Fig. 3a,), which likely represented the early separation of two sets of central mitosomes.

To further follow the separation of the central mitosomes, the cells were co-labeled for centrin, a basal body marker [49]. After their division the mitosomes remained associated with the basal bodies throughout the course of mitosis (Fig. 3b).

The prominent character of the central mitosomes was tested by the expression of a synthetic linker composed of the outer mitosomal membrane protein GiMOMP35 at the N-terminus [33], a central HA-tag, and the C-terminal SNARE protein GiQb4, which has been suggested to participate in membrane fusions on the cell periphery [50]. The topology of the construct was designed to dislocate the mitosomes by linking them to the peripheral endomembrane vesicles (Fig. 3c). Indeed, the expression of the synthetic linker dramatically perturbed the overall distribution of the mitosomes. Of about 40 peripheral mitosomes, only several large structures remained. These structures were positive for the synthetic linker and very likely represented mitosomal aggregates induced upon the linker expression (Fig. 3c). However, the central mitosomes remained largely unaffected by the expression of the linker (Fig. 3c). This could be explained by the association of the central mitosomes with the karyomastigont (structural complex of the basal bodies and the nuclei), which minimized the effect of the linker expression. Moreover, the linker also induced rearrangement of the ER network as documented by co-labeling by the ER marker protein, protein disulfide isomerase 2 (PDI2) [51]. Notably, the co-localization of the mitosome- and the ER-specific markers suggests that chimeric compartments may have been formed in these cells.

*G. intestinalis* undergoes DNA replication and nuclear division during the process of encystation, when tetranucleated 16 N cysts are formed [52]. To follow mitosomal dynamics during encystation, *G. intestinalis* cells were induced to encyst in vitro, and the cells were then fixed and immunolabeled. Similarly to mitotic trophozoites, the encysting cells were found to contain elongated mitosomes that often adopted a dumbbell shape, suggesting that mitosomes divide during encystation (Fig. 2b). Later encystation stages with the characteristic oval shape of the cyst were devoid of dividing mitosomes. However, these cells contained approximately twice as many mitosomes as the trophozoites (Fig. 2c, d). Collectively, these data show that, in addition to two pairs of nuclei, *G. intestinalis* cysts contain a double set of mitosomes, which enable the parasite to undergo rapid cell division during excystation in a new host.

### The single dynamin-related protein in *G. intestinalis* is not involved in mitosomal division

Mitochondrial division is mediated by dynamin-related proteins [53] or by the ancestral bacterial FtsZ-based machinery [18]. Moreover, the actin cytoskeleton was recently found to participate in mitochondrial division, possibly by inducing initial mitochondrial constrictions [54]. Thus, the roles of *G. intestinalis* dynamin-related protein (GlDRP) [55] and actin (GiActin) [56] in mitosomal division were investigated.

**Fig. 4:**
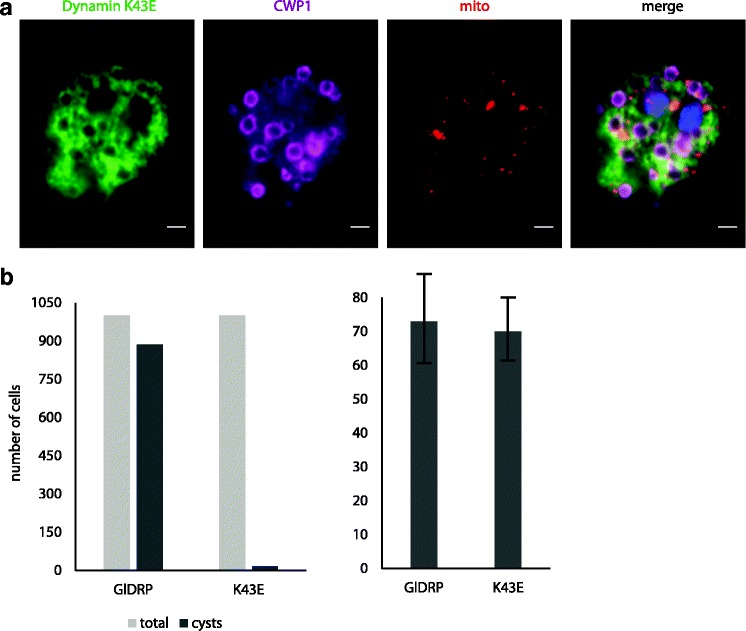
The single dynamin-related protein of *G. intestinalis* (*GiDRP*) is not involved in the division of mitosomes. **a** *G. intestinalis* cells expressing human influenza hemagglutinin (*HA*)-tagged K43E GlDRP were immunolabeled using anti-HA antibody (*green*), anti-GL50803_9296 antibody *(red*), and anti-CWP1 antibody (*magenta*). One layer of the whole Z-stack is shown. Scale bar, 2 μm. **b** *G. intestinalis* trophozoites were subjected to in vitro encystation and the number of formed cysts and the number of mitosomes within these cells were determined. For the latter, 50 encysting cells were used for the calculation. The error bars depict the standard deviations

*G. intestinalis* cells were transformed with a plasmid carrying HA-tagged GlDRP. In addition to the mitosomal and ER markers, the mitotic trophozoites were immunolabeled with the anti-HA antibody (Additional file 6). Most of the cellular dynamin was localized to the cytoplasmic membrane, where it takes part in the endosomal-lysosomal system of the peripheral vacuoles [55, 57]. However, there was no direct indication that GlDRP plays a role in mitosomal division. To further examine the possible role of GlDRP in mitosomal division, an HA-tagged, K43E-mutated version of GlDRP was introduced into *G. intestinalis* (Fig. 4a, Additional file 7). This mutation abolishes GTPase activity and causes a dominant negative effect in *G. intestinalis* [55]. Provided that encystation of *G. intestinalis* also involves mitosomal division, the K43E GlDRP was cloned behind the promoter region of cyst wall protein 1, expression of which is induced upon the encystation stimuli. As reported previously [55], the presence of K43E GlDRP resulted in the inability of the trophozoites to complete encystation (Fig. 4b). This phenotype supported the establishment of a dominant negative effect of K43E GlDRP in *G. intestinalis*. However, the affected encysting cells contained twice as many mitosomes as trophozoites. This strongly suggests that GlDRP is not involved in the division of mitosomes (Fig. 4b).

It has previously been shown that GiActin localizes to the axonemes and flagella, nuclei and the cortex of the trophozoites [56]. An inspection of the mitotic cells showed no association between the dividing mitosomes and GiActin (Additional file 8).

### The mitosomes associate with the endoplasmic reticulum

Dynamin-related proteins are not the only effectors of mitochondrial division. Recent data from mammalian and yeast cells have revealed the fundamental role of the ER–mitochondria connections in the dynamics of the mitochondrial network and the positioning of the mitochondrial division sites [8–10]. So far only limited data are available about whether such connections are present outside the supergroup of Opisthokonta, where animals and fungi belong. We tested if such associations also occur in *G. intestinalis*, which belongs to the Excavata [58].

**Fig. 5:**
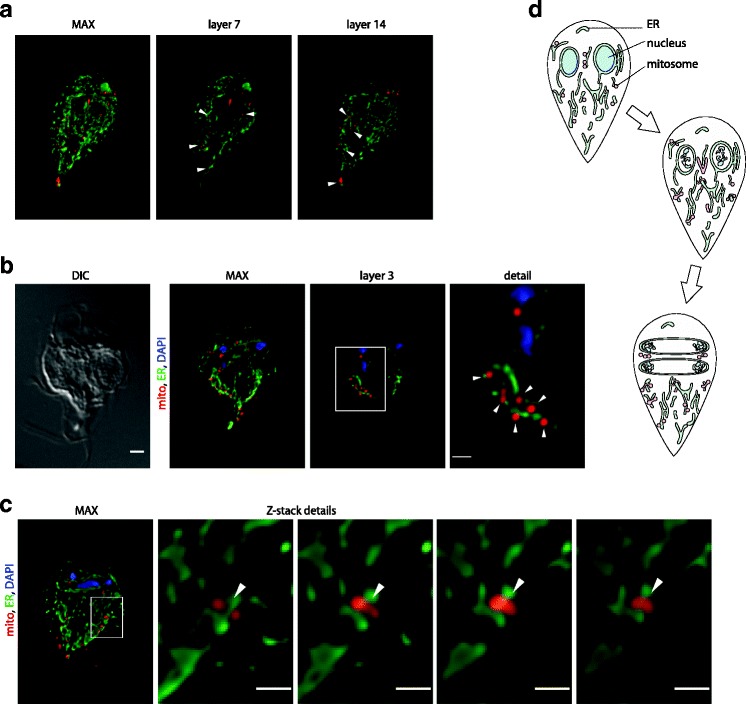
Mitosomes associate with the endoplasmic reticulum (*ER*) throughout the life cycle. **a** *G. intestinalis* trophozoites were fixed and immunolabeled using anti-GL50803_9296 (*red*) and anti-PDI2 antibodies (*green*) and observed using structured illumination microscopy (SIM). The maximal projection of the Z-stack of SIM images and selected Z-layers are shown. **b** *G. intestinalis* trophozoites enriched for mitotic cells by albendazole treatment were fixed and immunolabeled using anti-GL50803_9296 (*red*) and anti-PDI2 antibodies (*green*). The nuclei were stained with DAPI (*blue*). The images represent deconvolved maximal projections of the Z-stacks. Corresponding differential interference contrast (*DIC*) images are shown. Scale bars, 2 μm and 0.5 μm. **c** Ring-like mitosomal structures around the ER tubules. **d** Schematic representation of the ER–mitosome association and the mitosomal division synchronized with mitosis in *G. intestinalis*: *top*, the interphase cell with no observable mitosomal dynamics; *middle*, upon entry into mitosis, central and peripheral mitosomes start to divide; *bottom*, division of the central mitosomes completes during prophase as the divided organelles segregate along with the divided basal bodies to the opposite spindle poles. The peripheral organelles continue to divide throughout all mitotic stages

In order to visualize the distribution of the ER and the mitosomes, interphase trophozoite cells were co-labeled for the ER marker PDI2 [51] and the mitosomal marker GL50803_9296 [33]. The double labeling revealed a very close association between the ER tubules and the vast majority of the mitosomes in every cell (Fig. 5a). When compared to the mitochondrial networks of mammals and yeasts, the association appears even more prominent owing to the vesicular morphology of the mitosomes.

The two organelles remained associated during mitosomal division (Fig. 5b). Moreover, the dividing mitosomes elongated along the ER tubules, which indicates that the ER may serve as a platform for mitosomal division (Fig. 5c, d).

### The mitosome-associated endoplasmic reticulum is enriched for long-chain fatty acid CoA ligase 4

Several different molecular tethers mediate the association between the ER and the mitochondria. Fungi employ an ER–mitochondria tethering complex known as ERMES (ER–mitochondria encounter structure) consisting of four different components: Mdm10 and Mdm34 in the mitochondrial membrane, and the cytosolic Mdm12 and Mmm1 in the ER [11]. Analogous interactions seem to be mediated by the recently described ER membrane protein complex (EMC) [59] and Lam6 protein [13, 14], whose unifying function is interorganellar lipid transport. Animal mitochondria were shown to rely on the interactions between mitofusin 2 anchored in both the ER membrane and the outer mitochondrial membrane [15]. However, the function of this interaction has recently been questioned [16]. Importantly, all these structures have limited evolutionary distributions and none of them is present in metamonads, including *G. intestinalis* [60, 61]. In addition, several proteins are enriched in the so-called mitochondria-associated membranes (MAMs), a specific region of the ER, which comes into contact with mitochondria and mainly accommodates lipid and fatty acid metabolic enzymes [62].

**Fig. 6:**
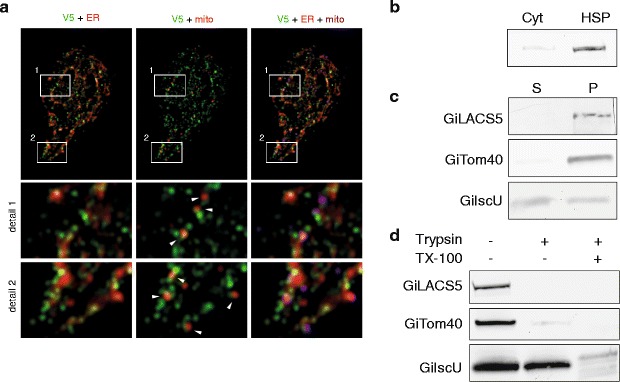
GiLACS4 populates the endoplasmic reticulum (*ER*)–mitosome contact sites. **a** *G. intestinalis* cells expressing V5-tagged GiLACS were fixed and immunolabeled using anti-V5 tag, anti-GL50803_9296, and anti-PDI2 antibodies. *Left*: V5 in *green* and PDI2 in *red*; *Middle*: V5 in *green* and GL50803_9296 in *red*; *Right*: V5 in *green*, PDI2 in *red* and GL50803_9296 in *magenta*. The cells were observed by structured illumination microscopy (SIM). The *arrows* indicate spots where the mitosomal signal meets the V5-tagged GiLACS4. **b** The cells were fractionated and the high-speed pellet (*HSP*) and cytosolic fraction were immunolabeled with anti-V5 antibody. **c** The HSP fraction was subjected to sodium carbonate extraction and the resulting fractions immunolabeled with anti-V5 (GiLACS4), anti-IscU, and anti-Tom40 antibodies. S - soluble fraction, P - membrane bound fraction. **d** The HSP fraction was treated with trypsin with or without the presence of 1% Triton. The samples were immunolabeled with anti-V5 (GiLACS4), anti-IscU, and anti-Tom40 antibodies

**Fig. 7:**
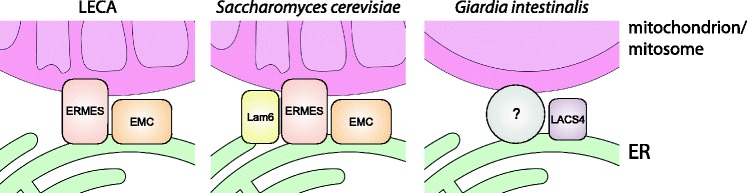
The evolution of mitochondrial dynamics in Excavata. A schematic representation of the occurrence of the mitochondrial fission and fusion machinery and the endoplasmic reticulum (*ER*)–mitochondria tethering complexes. The patchy phylogenetic distribution of FtsZ-based machinery [19] suggests that dynamin-based division of mitochondria appeared later in the evolution of eukaryotes and was not present in the last eukaryotic common ancestor (*LECA*). While both systems can be found in Excavata, *G. intestinalis* does not use either of them for the division of mitosomes. The lack of observable mitochondrial fusion and the responsible molecular machinery in Excavata indicates that the lack of fusion is an ancestral trait. The components of both recently described ER-tethering complexes can be found in all five supergroups of eukaryotes and thus they were likely present in the LECA [76]. Despite the secondary loss of these complexes, *G. intestinalis* mitosomes maintain association with the ER throughout the cell cycle, indicating the presence of yet unknown tethering mechanisms, perhaps including lipid metabolism enzymes such as LACS4. *ERMES* endoplasmic reticulum–mitochondria encounter structure, *EMC* endoplasmic reticulum membrane protein complex

Of the 21 known MAM marker proteins summarized in [62], our bioinformatic searches revealed a single candidate in the *G. intestinalis* genome: long-chain acyl-CoA synthetase 4, hereafter referred to as GiLACS4 [63]. GiLACS4 expressed with a C-terminal V5 tag localized to specific regions of the ER network (Fig. 6a). Importantly, GiLACS4 was also localized proximal to the mitosomes (Fig. 6a). Accordingly, the protein was present in the high-speed pellet fraction, which was enriched for both the ER and the mitosomes (Fig. 6b). Upon sodium carbonate treatment, GiLACS4 was retained in the pellet fraction, which indicates its insertion into the membrane (Fig. 6c). However, on trypsin treatment, the protein was exposed to the cytoplasm (Fig. 6d). Altogether, these data suggest that the mitosome–ER contact sites are occupied by the fatty acid activating enzyme, GiLACS4 (Fig. 7).

## Discussion

Mitosomes represent one of the most derived forms of mitochondria and are found in diverse anaerobic eukaryotes [25, 64]. During the course of mitochondrial evolution, the proteome of the mitosomes has shrunk to just a handful of proteins [27], whose sole role is the biosynthesis of iron–sulfur clusters [29, 33]. The mitosomes are devoid of the mitochondrial genome and cristae but have retained two organellar membranes. The stable number of mitosomes in *G. intestinalis* trophozoites indicates that their inheritance must be a controlled process, although alternate stochastic scenarios have also been suggested [32].

Mitochondrial dynamics, as studied in detail in fungal and animal cells, are controlled by dedicated molecular machineries governing both fusion and fission [1]. However, information on the mitochondrial dynamics outside Opisthokonta is scarce.

One of the striking characters of mitosomal dynamics is the synchrony between mitosis and mitosomal division. In our experiments, we have shown that both the central and the peripheral mitosomes divide exclusively during mitosis.

Earlier reports showed that the central mitosomes localize near the basal bodies and the axonemes between the two nuclei [47]. The division of this subpopulation of mitosomes occurs only in prophase and the daughter organelles then follow the separation of the chromosomes to the opposite spindle poles. The privileged localization of the central mitosome suggested that they may represent “germline” organelles, of which the peripheral organelles are derived upon mitosis [32, 65]. However, we show that the peripheral organelles also divide simultaneously during mitosis, including mitosis during encystation. This suggests that a mitosis-dependent signal for mitosomal division must exist in *G. intestinalis.*

Such overall harmonization of mitosomal division and mitosis has not been reported, to our knowledge, in any other eukaryote. In several instances a functional link between mitochondrial division and the cell cycle has been demonstrated, including for the mitochondria of kinetoplastids [66] and apicomplexans [67]. However, these organisms carry just a single mitochondrion, which, in the case of kinetoplastids, is even physically connected to the basal body of the flagellum [66]. Analogous behavior can be expected in other protists that carry a single mitochondrion, such as jakobids [68], where the organelle is often localized next to the cell nucleus.

What is the functional meaning of such synchronized division? We propose that the lack of mitosomal dynamics and the synchronized mitosomal division actually represent two sides of the same coin. The absence of dynamics in the interphase cell disqualifies the stochastic segregation of the organelles. Thus, harnessing the mitosomal and the nuclear division allows the cell to control the organelle number just before cytokinesis.

During the course of evolution, the FtsZ-based division machinery of the bacterial ancestor of mitochondrion has disappeared from most of the eukaryotes and has been replaced by the scission machinery driven by dynamin-related proteins. However, certain organisms from all supergroups of eukaryotes have preserved this ancestral division complex [19, 69], which suggests that the transition to the eukaryote-specific dynamin-based machinery occurred independently on numerous occasions. The group of Excavata to which *G. intestinalis* belongs comprises a great diversity of protists with a variety of mitochondrial forms, ranging from the single reticulate mitochondrion of kinetoplastids to the anaerobic vesicular forms known as hydrogenosomes and mitosomes of metamonads. So far, the division machinery has been characterized to some detail in mitochondria of *Trypanosoma brucei* [20, 21] and hydrogenosomes of *Trichomonas vaginalis* [22]. Here, dynamin-related proteins have been shown to participate in organelle division. Similarly to *Trypanosoma brucei*, the *G. intestinalis* genome encodes only for a single dynamin-related protein (GlDRP). This protein has been shown to function during the encystation process [55]. Indeed, we could show that its function is necessary for the completion of encystation, yet the presence of the dominant negative form of GlDRP did not affect the division of mitosomes, which is in contrast to the recent finding of Rout et al. [70]. However, while our data suggest that dynamin-related proteins are not involved in mitosomal division, it is also possible that the level of the dominant negative form of dynamin capable of preventing encystation is not sufficient to interfere with mitosomal division. Considering that neither of the outer membrane DRP1 recruitment factors, such as Mff and Fis1 [2], is present in the *G. intestinalis* genome, the responsible mitosome division machinery remains entirely unknown. This also includes the absence of GiActin at the dividing organelles.

Instead, mitosomes maintain a vital connection to the ER throughout the cell cycle and the association becomes more prominent during mitosomal division. While the nature of the connection is unknown, we have shown that the ER–mitochondria interface is populated by the fatty acid activating enzyme LACS4. Thus, it is likely that the ER–mitosomal association enables lipid and/or fatty acid transport between the compartments as documented for the mitochondria of animals and fungi [12, 62]. Unfortunately, direct biochemical characterization of the mitosome-associated ER membrane fraction is not feasible owing to the lack of procedures enabling specific organelle purification [27]. A recently developed technique involving in vivo biotinylation and cross-linking of the target protein [33] enables the bypassing of such experimental limitations, although optimization toward the native purification conditions will be required.

The bridging complexes between mitochondria and the ER include the ERMES and the EMC complexes [11, 59]. The ERMES complex was originally described in *Saccharomyces cerevisiae* as the first bona fide structure specialized in tethering the mitochondrial and ER membranes [11]. The complete set of four ERMES components can be found across all supergroups of eukaryotes, although is missing in most Excavata species [60]. The EMC is more conserved among eukaryotes, but it is missing in all metamonads including *G. intestinalis* and *Trichomonas vaginalis* [61]. Considering that both the ERMES and the EMC were likely present in the last eukaryotic common ancestor (LECA), it is highly probable that they were lost in *G. intestinalis* and perhaps all metamonads.

Our data suggest that the overall dynamics of mitosomes in *G. intestinalis* is secondarily reduced down to organelle division. The mitosomes do not manifest any observable dynamics in the interphase cells, as their number and morphology remained constant upon metabolic stress induced by 5-nitroimidazole or under iron deficiency, which affects their single metabolic function of the iron–sulfur clusters formation. By contrast, highly enlarged hydrogenosomes appear in *Trichomonas vaginalis* treated with 5-nitroimidazole (metronidazole) and other drugs [39], and the organelles undergo distinct transformation upon the lack of iron ions [71].

Interestingly, mitosomal fusion was not observed in our experiments. While it is possible that the organelles fuse under very low frequency, the process is not efficient enough to provide a homogeneous population of mitosomes. Moreover, the *G. intestinalis* genome does not encode orthologs of the mitochondrial membrane fusion proteins identified in opisthokonts [6, 7]. It is, however, important to note that other lineages of eukaryotes, including Archaeplastida (e.g., *Arabidopsis thaliana*), which exhibit mitochondrial fusion, also do not rely on opisthokont machinery [72].

Nevertheless, the absence of mitochondrial fusion seems to be common to the whole supergroup of Excavata, as it has not been observed in any studied species so far. Neither have the orthologs of components governing the mitochondrial membrane(s) fusion been identified. Taken together, it is plausible that mitochondrial fusion appeared independently in other lineages of eukaryotes outside Excavata, perhaps employing different, lineage-specific molecular machinery. Whether the lack of mitochondrial fusion concerns also the LECA awaits further and more complex comparative analyses.

Although simple in their shape and function, the mitosomes of *G. intestinalis* show unique and sophisticated dynamics, which seem to be a mosaic of evolutionarily conserved traits and lineage-specific inventions. Nevertheless, an understanding of the molecular machinery responsible for mitosomal division and its synchrony as well as the nature of the ER–mitosome connections poses exciting possibilities for future research.

## Conclusion

The mitochondria of animals and fungi undergo constant cycles of division and fusion during the cell cycle. Here, we show that the minimalist MROs known as mitosomes have dramatically simplified their dynamics. In the anaerobic protist *G. intestinalis*, mitosomes divide only during mitosis and remain steady during interphase. In contrast to mitochondria, we propose that mitosomes do not fuse but, similar to mitochondria, maintain a close connection to the ER. We propose that harnessing the nuclear and mitosomal division is a strategy evolved to bypass the lack of mitochondrial fusion.

## Methods

### *G. intestinalis* cultivation and transfection

*G. intestinalis* cells (strain WB) were cultured in TYI-S-33 medium supplemented with 10% heat-inactivated adult bovine serum (GE Healthcare, Chicago, IL, USA), 0.1% bovine bile (Sigma-Aldrich, St. Louis, MO, USA), and appropriate antibiotics at 37 °C. Cells were electroporated using a previously published modified protocol [73]. Briefly, 300 μL of cell culture at an approximate concentration of 3.3 × 10^7^ cells/mL was electroporated with 50 μg of a circular plasmid using a Bio-Rad Gene Pulser (BioRad, Hercules, CA, USA) with the exponential protocol (350 V, 1000 μF, 750 Ω). Transformants were maintained under selection with 57 μg/mL of puromycin (Gold Biotechnology, St. Louis, MO, USA) and/or 0.56 mg/mL G418 (Gold Biotechnology, St. Louis, MO, USA). For iron-starvation experiments, cells were incubated in TYI-S-33 medium without ferric ammonium citrate and supplemented with 2,2’-dipyridyl (Sigma-Aldrich, St. Louis, MO, USA) to a final concentration of 300 μM. The cell culture was maintained for several passages under these conditions.

### Enrichment of mitotic cells

Two approaches for cell synchronization were tested: the starvation [42] and the albendazole-dependent [43] methods. Both methods provided the same results concerning the mitosomal dynamics. However, the albendazole treatment had a much greater effect on cell synchrony and therefore was used in the study. Trophozoites from the late log phase were incubated in growth medium supplemented with 100 ng/mL of albendazole (Sigma-Aldrich, St. Louis, MO, USA) for 6 h at 37 °C [43]. After incubation, the albendazole-affected unattached cells were discarded and the unaffected adherent pre-mitotic cells were washed twice with pre-warmed, fresh, drug-free medium and then detached from the tube by cooling on ice for 10 min. The cells were then allowed to proliferate on slides in the drug-free conditions for 9–14 min, fixed, and permeabilized as described below.

### *G. intestinalis* encystation

In vitro encystation was performed as previously described [74]. Briefly, log-phase cells were incubated at 37 °C for 18 h in TYI:GS3 media at pH 7.8 that was supplemented with 5 mg/mL bovine bile (Sigma-Aldrich, St. Louis, MO, USA) and 0.546 mg/mL lactic acid (Sigma-Aldrich, St. Louis, MO, USA). After incubation, the medium was replaced with TYI-S-33 medium and the cells were incubated at 37 °C for several hours until they started to produce cysts. The cysts were then fixed with 1% paraformaldehyde for 30 min at room temperature and placed on slides.

### Immunofluorescent labeling

For the immunofluorescence, trophozoites were incubated on slides in TYI-S-33 medium for 15 min at 37 °C, fixed in ice-cold methanol for 5 min, and permeabilized in ice-cold acetone for 5 min. The blocking and the immunolabeling steps were all performed in a humid chamber using a solution of 0.25% bovine serum albumin (BSA)(Sigma-Aldrich, St. Louis, MO, USA), 0.25% fish gelatin (Sigma-Aldrich, St. Louis, MO, USA), and 0.05% Tween 20 (Sigma-Aldrich, St. Louis, MO, USA) in phosphate-buffered saline (PBS) for 1 h each. The primary antibodies used in this work included rat anti-HA monoclonal IgG antibody (Roche, Basel, Switzerland, 1:1000 dilution), mouse anti-actin polyclonal antibody (gift from Alex Paredez, University of Washington, 1:250 dilution) [56], rabbit anti-GL50803_9296 polyclonal antibody (1:2000 dilution) [33], and mouse anti-GiPDI2 polyclonal antibody (a gift from Adrian Hehl, University of Zurich, 1:2000 dilution) [51]. The secondary antibodies included Alexa Fluor 488-conjugated goat anti-rat monoclonal IgG antibody (Invitrogen, Eugene, OR, USA, batch number 1476598, cat. number A-21208, RRID: AB_141709; 1:1000 dilution), Alexa Fluor 647-conjugated goat anti-mouse monoclonal IgG antibody (Invitrogen, Eugene, OR, USA, batch number 1511346, cat. number A-21235, RRID: AB_141693; 1:1000 dilution), Alexa Fluor 594-conjugated goat anti-rabbit monoclonal IgG antibody (Invitrogen, Eugene, OR, USA, batch number 1454437, cat. number, A-21207, RRID: AB_141637; 1:1000 dilution), and Alexa Fluor 488-conjugated goat anti-mouse monoclonal IgG antibody (Invitrogen, Eugene, OR, USA, batch number 1562298, cat. number A-21202, RRID: AB_141607; 1:1000 dilution). Three 5-min washes in PBS were performed after each immunolabeling step. Slides were mounted in Vectashield (Vector Laboratories, Burlingame, CA, USA) containing DAPI.

The cysts were fixed in 1% paraformaldehyde for 30 min at 37 °C and spun down at 1000 × *g* for 5 min at room temperature. The cysts were then washed in 1× PEM buffer (100 mM PIPES pH 6.9, 1 mM EGTA, and 0.1 mM MgSO_4_), resuspended in 1× PEM buffer, and placed on cover slips. Cell permeabilization was performed using 0.2% Triton X-100 (Sigma-Aldrich, St. Louis, MO, USA) for 20 min. The cover slips were then washed three times with 1 mL of 1× PEM and incubated with anti-GL50803_9296 rabbit polyclonal antibody (1:2000 dilution) in 1× PEMBALG [100 mM PIPES pH 6.9, 1 mM EGTA, 0.1 mM MgSO_4_, 1% BSA, 0.1% NaN_3_, 100 mM lysine, and 0.5% cold-water fish skin gelatin (Sigma-Aldrich, St. Louis, MO, USA)] for 1 h. After three 5-min washes in 1× PEM, the slides were incubated with Alexa Fluor 594-conjugated goat anti-rabbit IgG antibody in 1× PEMBALG for 1 h. After three 5-min washes in 1× PEM, the slides were mounted in Vectashield containing DAPI.

For live-cell imaging experiments, trophozoites expressing an IscU-Halo tag fusion product [31] were incubated in growth medium supplemented with the TMR Halo ligand (Promega, Madison, WI, USA, 1:1000 dilution) for 1 h at 37 °C. To wash away unbound TMR ligand, the cells were washed twice with pre-warmed fresh medium and incubated for 30 min at 37 °C. After incubation, the cells were placed on ice for 10 min. The cells were then transferred to a microscope dish and observed using a confocal microscope.

### Imaging

Static images were acquired on an Olympus IX-81 microscope using a UPlanSApo 100×/1.4 numerical aperture (NA) oil-immersion objective. Z-stacks of images ranging between 0.23 and 0.25 μm were captured using an ORCA C4742-80-12AG monochromatic CCD camera (Hamamatsu, Shizuoka, Japan). Fluorescence was excited with a xenon arc burner-containing MT20 illumination system (Olympus, Tokyo, Japan), and emitted light was collected through a multiband emission filter. Imaging was controlled with the Olympus Cell-R software. Images were deconvolved using SVI Huygens software with the CMLE algorithm. Maximum intensity projections and brightness/contrast corrections were performed in FIJI ImageJ.

Structured illumination microscopy (SIM) imaging was also performed on a commercial 3D N-SIM microscope (inverted Nikon Eclipse Ti-E, Nikon, Tokyo, Japan) equipped with a Nikon CFI SR Apo TIRF objective (100× oil, NA 1.49). A structured illumination pattern projected into the sample plane was created on a diffraction grating block (100 EX V-R 3D-SIM) for laser wavelengths of 488, 561, and 647 nm. Excitation and emission light was separated using filter cubes with the appropriate filter sets SIM488 (excitation 470–490 nm, emission 500–545 nm), SIM561 (excitation 556–566 nm, emission 570–640 nm) and SIM647 (excitation 590–650 nm, emission 663–738 nm). Emission light was projected through a 2.5× relay lens onto the chip of an electron-multiplying charge-coupled device (EMCCD) camera (Andor iXon Ultra DU897, 10 MHz at 14-bit, 512 × 512 pixels). Three-color Z-stacks (Z-step: 120 nm) were acquired using NIS-Elements AR software (Laboratory Imaging). Laser intensity, electron-multiplying gain, and camera exposure time were set independently for each excitation wavelength. The intensity of the fluorescence signal was held within the linear range of the camera. Fifteen images (three rotations and five phase shifts) were recorded for every plane and color. SIM data were processed in NIS-Elements AR. Before sample measurement, the symmetry of the point spread function was checked with 100 nm red fluorescent beads (580/605, Carboxylate-Modified Microspheres, Molecular Probes, Eugene, OR, USA) mounted in Prolong Diamond Antiface Mountant (Molecular Probes, Eugene, OR, USA), and optimized by adjusting the objective correction collar. The live-imaging differential interference contrast (DIC) microscopy time series and confocal fluorescence images were acquired on an Olympus IX-81 microscope equipped with a Yokogawa CSU-X1 spinning disc unit and an Andor DU-897 EMCCD camera using an UPlanSApo 60×/1.35 NA oil-immersion objective (Olympus, Tokyo, Japan). Fluorescence was excited with a 561-nm laser (Coherent Inc., Santa Clara, CA, USA) and collected through a multiband emission filter (Semrock FF01-440/521/607/700). Typical Z-stacks were captured with a 0.5-μm Z-axis step. After imaging, images were processed in FIJI ImageJ software.

### Plasmid construction and cloning

The *G. intestinalis* dynamin gene (GL50803_14373) and 250 base pairs of its 5′ untranslated region (UTR) were amplified together from *G. intestinalis* genomic DNA using the primers 5′-CATGGATATCACAACGAGGCTTTAAGCC-3′ and 5′-CATGATGCATGTCCTTCTTGGCAAGGTC-3′, which contain EcoRV and NsiI restriction sites, respectively. The resulting product was cloned as an EcoRV/NsiI fragment into an EcoRV/PstI-linearized pTG vector. The *G. intestinalis* centrin gene (GL50803_6744) and 300 bp of its 5′ UTR were amplified together from *G. intestinalis* genomic DNA using the primers 5′-CATGGATATCTGCCCATGGCTATGGTGT-3′ and 5′-CATGCTGCAGATAGAGGGACGTGCGGCG-3′, which contain EcoRV and PstI restriction sites, respectively. The resulting product was cloned as an EcoRV/PstI fragment into an EcoRV/PstI-linearized pTG vector.

To generate mutant K43E dynamin (GL50803_14373), the mutation was introduced by site-directed mutagenesis using the primers 5′-CATGACGCGTTATGTCTCAGATAGACAAG-3′ and 5′-CTCCAAAACCGATGACTCTCCCGCAGA-3′, and 5′-TCCCAATCTGCGGGAGAGTCATCGGTT-3′ and 5′-CATGGCGGCCGCTCCTTTCTTGGCAAGGTC-3′. The resulting product was cloned as an MluI/NotI fragment into a MluI/NotI-linearized pPAC vector.

The N-terminally HA-tagged gene for GiQb4 was amplified from *G. intestinalis* genomic DNA using primers 5′-CTAGGGATCCATGTACCCATACGATGTTCCAGATTACGCTGAAGAGATAGAATGTTCACTCA-3′ and 5′-GCTAGTCGACTCAATATCTGATCTCTGA-3′ containing BamHI/SalI restriction sites, respectively. Resulting product was cloned as a BamHI/SalI fragment to a BamHI/XhoI-linearized pONDRA plasmid containing the gene for GiMOMP35 [27].

### Cell fractionation

*G. intestinalis* cells were collected in ST buffer containing protease inhibitors TLCK and Leupeptine. The cells were sonicated by 1-s pulses at amplitude 40 until all the cells were completely lysed. The lysate was centrifuged at 2.680 *× g* for 20 min at 4 °C. The supernatant was centrifuged at 180.000 *× g* for 30 min at 4 °C. The resulting supernatant was considered as the cytosolic fraction while the pellet were considered as the high-speed pellet (HSP) fraction.

### Sodium carbonate extraction

For sodium carbonate extraction, 50 μL of the cellular HSP fraction was mixed with 200 μL of freshly made 100 mM Na_2_CO_3_ (pH 11) and incubated on ice for 30 min. The sample was mixed vigorously every 2 min. After incubation, the sample was centrifuged at 100,000 *× g* for 30 min at 4 °C. The supernatant was then mixed with TCA (trichloracetic acid) to a final concentration of 20% and incubated on ice for 30 min. The pellet (1) was kept on ice. After incubation with TCA, the supernatant was centrifuged at 30,000 *× g* for 10 min at 4 °C. The pellet (2) was rinsed with 0.5 mL of ice-cold acetone and centrifuged at 30,000 *× g* for 10 min at 4 °C. Both pellets were mixed with 50 μL of 1 × SDS (sodium dodecyl sulfate) sample buffer and incubated at 95 °C until dissolved.

### Trypsin treatment

For treatment with trypsin, 20 μL of the cellular HSP fraction containing 150 μg of proteins was mixed with trypsin (5 mg/mL) or trypsin and 1% Triton X-100 as follows: (1) 20 μL HSP + 30 μL SM (sucrose, MOPS) buffer; (2) 20 μL HSP + 28 μL SM buffer + 2 μL trypsin; and (3) 20 μL HSP + 23 μL SM buffer + 5 μL Triton X-100.

All samples were incubated for 10 min at 37 °C and boiled in 50 μL of 1 × SDS sample buffer at 95 °C for 5 min.

### Determination of enzymatic activities

All enzyme activities were assayed spectrophotometrically at 25 °C. The activity of PFO was assayed as the rate of methyl viologen reduction monitored at 600 nm. The assay was performed under anaerobic conditions using pyruvate as a substrate for PFO as described in [75].

## Additional files

Additional file 1: Dividing mitosomes in mitotic *G. intestinalis. G. intestinalis* culture expressing IscU-Halo was enriched for mitotic trophozoites by albendazole treatment (100 ng/mL) for 6 h at 37 °C. The cells were washed twice in warm medium and stained by Halo-TMR ligand and observed under a microscope. The images are representative of the sequence submitted as movie files (Additional files 2, 3, and 4). (EPS 7706 kb)

Additional file 5: Peripheral mitosomes divide during all stages of mitosis. (**A**) *G. intestinalis* culture was enriched for mitotic trophozoites by albendazole treatment (100 ng/mL) for 6 h at 37 °C. The cells were washed twice in warm medium and fixed, and the mitosomes were immunolabeled with an anti-GL50803_9296 antibody (red) and stained for nuclei with DAPI (blue). The image represents a deconvolved maximal projection of the Z-stack. Corresponding DIC images are shown. Scale bar, 2 μm. Arrowheads point at dividing mitosomes. (**B**) The number of mitosomes in particular stages of mitosis was determined using fixed cells. The data show a gradual increase in mitosome number during mitosis. Thirty cells of each mitotic stage were used for the statistics. The error bars represent the standard deviations. (EPS 4730 kb)

Additional file 6: Distribution of dynamin in mitotic *G. intestinalis* cells. *G. intestinalis* expressing HA-tagged GlDRP was enriched for mitotic trophozoites. The cells were immunolabeled using anti-GL50803_9296 antibody (red), anti-PDI2 antibody (magenta), and anti-HA antibody (green). Selected layers of the Z-stack are shown with the corresponding DIC image. Scale bar, 2 μm. (EPS 2840 kb)

Additional file 7: The expression of K43E GlDRP in *G. intestinalis.* The cell lysate of the encysting cells was probed for the presence of HA-tagged K43E GlDRP. The arrow points toward the expected size of the protein on the western blot. (EPS 3276 kb)

Additional file 8: Distribution of actin in mitotic *G. intestinalis. G. intestinalis* culture was enriched for mitotic trophozoites. (**A**) The cells were immunolabeled using anti-GL50803_9296 antibody (red) and anti-GiActin antibody (green). The image represents the deconvolved maximal projection of the Z-stack (MAX). (**B**) The cells were immunolabeled using the anti-PDI2 antibody (red) and anti-GiActin antibody (green). The images represent the deconvolved maximal projection of the Z-stack (MAX) and two selected layers. Corresponding DIC images are shown. Scale bar, 2 μm. (EPS 5063 kb)
